# Locked intramedullary femoral nailing without fracture table or image intensifier

**DOI:** 10.1007/s11751-011-0122-3

**Published:** 2011-11-13

**Authors:** Rajesh Rohilla, Roop Singh, Seema Rohilla, Narender K. Magu, Ashish Devgan, Ramchander Siwach

**Affiliations:** 1 Department of Orthopaedic Surgery, Paraplegia and Rehabilitation, Pt. B.D. Sharma PGIMS, 9 J/28, Medical Enclave, Rohtak, Haryana 124001 India; 2 Department of Radiodiagnosis, Pt. B.D. Sharma PGIMS, Rohtak, Haryana 124001 India

**Keywords:** Fracture, Femur, Image intensifier, Fracture table, Intramedullary nailing, Interlocking, Closed reduction technique

## Abstract

The present retrospective study aims to evaluate the outcome in 41 patients of femoral shaft fractures, who had closed intramedullary nailing in lateral decubitus position without fracture table or image intensifier. Mean age was 33.2 (range, 18–70) years. The cannulated reamer in proximal fragment (as intramedullary joystick) and Schanz screw in the distal fragment (as percutaneous joystick) were simultaneously used to assist closed reduction of the fracture without the use of image intensifier. Closed reduction was successful in 38 patients. Open reduction was required in 3 patients. Schanz screw was used for closed reduction in 12 patients. Average number of intra-operative radiographic exposures was 4.4. Two patients had exchange nailing using large diameter nails. One patient had nonunion. Angular and rotatory malalignments were observed in seven patients. We are of the opinion that the present technique is a safe and reliable alternative to achieve closed locked intramedullary nailing and is best suited to stable, less comminuted (Winquist–Hansen types I and II) diaphyseal fractures of the femur.

## Introduction

The femoral shaft fractures in adults are preferably treated with closed intramedullary nailing [1–4]. Closed reduction is a critical component of the procedure [5], and fracture table is used to generate longitudinal traction to achieve closed reduction and maintain the reduction during the operative fixation [3, 6–13]. The complications reported following use of traction on a fracture table are pudendal nerve palsy [14], well-leg compartment syndrome [15], and skin sloughs of the perineum [16]. Intramedullary nailing of femur without a fracture table has been reported [17–20]. Compared with fracture-table traction, manual traction for intramedullary nailing of isolated fractures of the femoral shaft has been shown to decrease operative time and improve the quality of the reduction [18, 21].

Nailing of the femur can be performed in both supine [4, 8–10, 21, 22] and lateral position [4, 17, 20, 22]. The supine position is physiologic and convenient to the anesthetist and is preferred if patient also have cervical spine injury, ipsilateral lower extremity fracture and severe pulmonary compromise [17, 23]. But access to greater trochanter is somewhat limited in supine position, particularly in large or obese patients in whom lateral position is preferred [23]. Fluoroscopy is used extensively during locked intramedullary nailing, which increases the intra-operative radiation exposure [5, 24]. Weil et al. [5] recently reported that computerized navigation has the potential for increasing precision in fracture reduction while minimizing fluoroscopic requirements, but this facility is not universally available. Even both facilities of an image intensifier and fracture table are difficult to come by in third-world countries [17]. This study aims to evaluate the outcome in 41 patients of femoral shaft fractures who had closed intramedullary nailing in lateral decubitus position without fracture table or image intensifier.

## Materials and methods

From March 2006 to October 2008, closed diaphyseal fractures of the femur (AO type 32) in 41 nonconsecutive patients were stabilized without the use of image intensifier on ordinary operation table in lateral decubitus position and were retrospectively evaluated. The study was approved by Institutional Review Board. Patients with pathological fractures, open fractures, severely comminuted fractures (AO type 32 C3), ipsilateral femoral fractures of proximal and distal segments (i.e., AO type 31 and 33) and ipsilateral tibial fractures were excluded from the study. Mean age of 41 patients (32 men and 9 women) was 33.2 years (range 18–70 years). Twenty-nine fractures were the result of road traffic accidents and 12 fractures were due to fall. After initial management in Accident and Emergency Department all patients were put on skeletal traction through upper tibial Steinman pin with weights of 7–12 kg till operation. As per AO classification, 13 patients had type A [A1 (*n* = 2), A2 (*n* = 9), A3 (*n* = 2)], 22 patients had type B [B1 (*n* = 4), B2 (*n* = 11), B3 (*n* = 7)], and 6 patients had type C [C1 (*n* = 3), C2 (*n* = 3)] fractures. All patients with type A fractures were grouped as group I (AO) (*n* = 13) and compared with patients with comminuted fractures of types B and C (group II (AO), *n* = 28). According to Winquist-Hansen (WH) classification [3], 20 fractures were type I; 11 were type II; four were type III; and six were type IV. Patients with moderate or no comminution according to Winquist–Hansen classification (types I and II) were grouped as group I (WH) (*n* = 31) and were compared with patients with severe comminution with type III and IV fractures (group II (WH), *n* = 10) to assess the effect of comminution. Operations were performed with in a mean of 5.9 days (range, 2–14 days) following trauma. First-generation intramedullary locked nails were used. To determine the approximate length of the nail before surgery, the distance from the tip of the greater trochanter to the intra-articular space of the knee on the patient’s uninjured side was measured and 20–30 mm was subtracted from it, preferring longer nail in distal one-third fractures of femur.

## Operative technique

### Fracture reduction and nail insertion

**Fig. 1:**
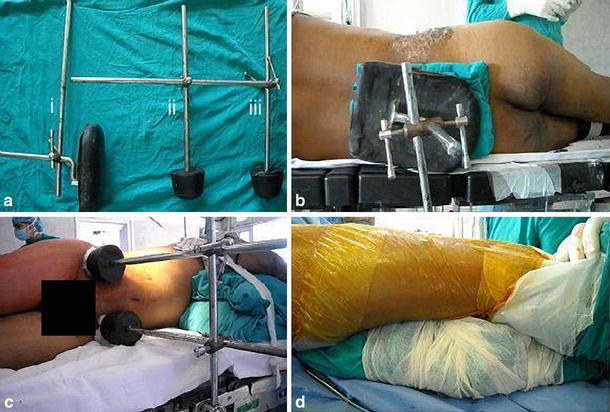
**a** Padded Posts used to stabilize the pelvis. Sacral post (*i*), conical anterior superior iliac spine posts (*ii* and *iii*). **b** Sacral post is applied on the lower back. **c** The two conical padded posts are applied in front at both anterior superior iliac spines in an oblique manner. **d** A soft pillow is placed between the legs to provide support and prevent sagging of the distal fragment

**Fig. 2:**
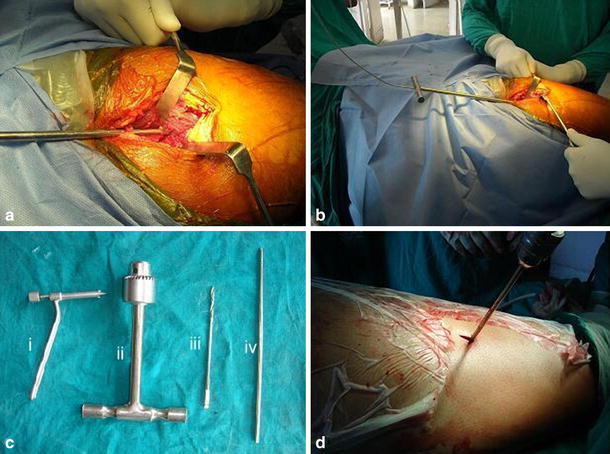
Surgical technique—**a** 8-mm straight stiff handheld reamer used to enlarge the proximal femoral canal; **b** insertion of cannulated reamer over guide wire; **c** instruments used to insert percutaneous Schanz screw to aid fracture reduction. (*i*) drill sleeve with trocar, (*ii*) T handle, (*iii*) 3.2-mm drill bit, (*iv*) 4.5-mm cortical Schanz screw, **d** insertion of the percutaneous Schanz screw

The patient is operated in the lateral decubitus with the fractured leg uppermost under general or regional anesthesia. The patient’s pelvis is stabilized in exact lateral position with the help of padded posts at two anterior superior iliac spines and at sacrum (Fig. 1a). First a sacral post on the lower back is applied (Fig. 1b). Then, the two conical padded posts are applied in front at both anterior superior iliac spines in an oblique manner (Fig. 1c). These posts prevent forward and backward bending of the patient at the level of pelvis and prevent secondary effect of pelvis mal-position. After prepping and draping of the patient, a soft pillow is placed between the legs to provide support and prevent sagging of the distal fragment (Fig. 1d). A 5–8-cm skin incision is made extending proximally from the greater trochanter. The tensor fascia lata and the abductor muscles are split along the incision down to the greater trochanter to expose the piriform fossa. The proximal femoral canal is entered through piriform fossa using a curved awl. Eight- and nine-mm straight stiff handheld reamers are used to enlarge the proximal femoral canal (Fig. 2a). A guide wire is inserted into the proximal fragment after removal of stiff reamer; 9-mm straight stiff handheld cannulated reamer is inserted over the guide wire in the proximal fragment (Fig. 2b). The cannulated reamer in the proximal fragment is used as intramedullary joystick to control the proximal fragment. Fracture is reduced with traction through skeletal pin. Movements of the guide wire should be gentle and are based on the grating feel of bony resistance produced by sliding of the guide wire along the inner cortical surface in the intramedullary canal of the distal fragment. In case of difficulty in guide wire insertion in the distal fragment, following maneuver can be tried to assist fracture reduction.

The instruments used in this maneuver are shown in Fig. 2c. A stab incision is given on the lateral aspect of distal fragment about 5-cm distal to the fracture site. A 5-mm drill sleeve (preferably with serrated end) is inserted up to the bone. A 3.2-mm drill bit is used to drill the near cortex of the bone in the distal fragment. A 4.5-mm Schanz screw is inserted through the drill sleeve into the near cortex of distal fragment (Fig. 2d). Now, the surgeon controls the distal fragment with the Schanz screw and the proximal fragment with the help of cannulated reamer to achieve fracture reduction, and the assistant inserts the guide wire through the cannulated reamer into the canal of distal fragment. Plain anteroposterior and lateral radiographs must be taken at this stage to confirm guide wire positioning in the distal fragment using portable X-ray machine*.* The reaming of the canal is performed up to the desired level. The reaming should be gentle and excessive force should never be used. Over reaming of 1.5 mm is necessary to prevent nail deformation while insertion. An interlocking nail of appropriate size is inserted. Intra-operative radiographs may be taken to assess fracture reduction, nail length, and alignment prior to locking.

### Locking

Distal locking can be performed using distal jig [17] or with nail over-nail technique [25]. Both techniques are image intensifier independent. Nail over-nail technique for distal locking [25] is as follows.

**Fig. 3:**
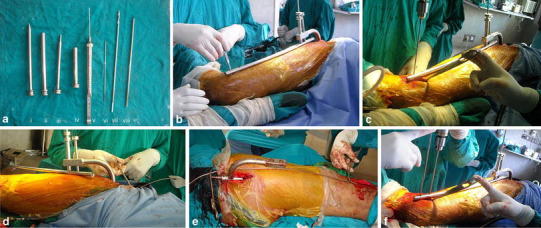
Surgical technique—**a** instruments used for nail–over-nail technique of distal locking (*i*) Trocar, (*ii*) Cannula, (*iii*) Trocar, (*iv*) Cannula, (*v*) Depth gauge, (*vi*) 2-mm K-wire, (*vii*) 4-mm drill bit, (*viii*) Steinnman pin. **b** A 2-cm incision at the site corresponding to the distal most screw of the second nail. **c** Drilling of the lateral cortex; **d** confirmation of the position of screw inside the nail hole with the help of ‘sounding technique’ using guide wire. **e** The length of the guide wire reached up to the screw further confirms the proper placement of the screw by comparing from outside. **f** Locking of the second distal (more proximal) screw

Figure 3 a shows the instruments used for the technique. Another nail of same length (can be of different diameter) is placed over the thigh along the longitudinal axis of the femur. Two trocars and cannulae are inserted through the holes of the proximal guide into the proximal holes of the second nail. Second nail is aligned along the longitudinal axis of the femur. A 2-cm incision is made down to the bone at the site corresponding to the distal most screw of the second nail (Fig. 3b). A 4.0-mm drill (same drill bit which is recommended for the interlocking screws) is passed through the distal most hole of the second nail and drilled into the lateral cortex (Fig. 3c). Second nail is withdrawn, and light is focused over the hole in the lateral cortex. A fine suction tip is used to aid visualization of the nail hole through the hole in the lateral cortex. It is usually possible to insert drill bit through the nail hole. A 2-mm K-wire is very useful to locate the nail hole. Nail can be manipulated axially and rotationally by 1–2 mm if required for inserting drill bit through the nail hole. The drill bit in the distal most hole of the nail is confirmed with a metallic feel or typical sound generated as the guide wire introduced from the proximal end of the nail hits the drill bit distally (Fig. 3d). After confirming drill bit in the distal most hole, opposite cortex is drilled and a depth gauge is used to measure the length of the locking screw. A locking screw of appropriate size is inserted in the distal most interlocking hole. Again, a guide wire is used to confirm the position of screw with the help of ‘sounding technique’ and the length of the guide wire reached up to the screw confirms the proper placement of the screw by comparing from outside (Fig. 3e). Now, second nail is again placed over the thigh and two trocars and cannulae are inserted through the holes of the proximal guide into the proximal holes of the second nail. A Steinman pin is passed through the distal most hole of the nail to the just inserted distal most screw. This stabilizes the second nail placed along the longitudinal axis of the femur. A 2-cm incision is made down to the bone at the site corresponding to the second distal screw of the second nail. A 4.0-mm drill is passed through the second distal hole of the second nail and drilled into the lateral cortex (Fig. 3f). Rest of the procedure to lock the second distal screw is same as for distal most screw.

The rotational alignment of the fracture is checked before the proximal locking. The patella should be facing parallel to the floor in neutral position with the knee in 90° flexed position, and proximal locking is performed using proximal interlocking guide.

Failure of the closed reduction can be overcome by open reduction of the fracture through a small incision (3–6 cm) at fracture site just to insert the guide wire into the distal fragment. A failure of the distal locking was recorded when more than one hole was drilled in lateral cortex during single distal interlocking. In case of failure, distal locking was achieved with free hand technique if image intensifier was available.

Fracture reduction was examined on the postoperative anteroposterior and lateral radiographs with the use of a goniometer to determine angulation in the coronal (varus–valgus) and sagittal (flexion–extension) planes. Angular malreduction was defined as more than 5° of angular deformity in either the coronal or sagittal plane [10, 18]. Shortening more than 1 cm was considered as malalignment [7]. For the assessment of malrotation in the present study, the anteversion of both fractured side and contra lateral side was determined by a standard computed tomography (CT) torsion study, and the difference between the two sides was evaluated. Patients were placed supine on the CT scan table, with their legs taped to the sides to prevent movement during the study. Three-mm cuts were made through the femoral neck region and condylar region of both femora simultaneously. Anteversion of the femur was defined as the angle between the axis of the femoral neck and the line drawn tangent to the posterior femoral condyles. The increase in anteversion represented internal rotation of the distal fragment, whereas decrease in anteversion represented external rotation of the distal fragment. The difference in anteversion more than 15° was considered as malrotation [6, 7, 13, 26]. All the measurements to assess radiographic alignment were obtained by the independent radiologist, one of the authors of present study (SR).

## Statistical analysis

Data were analyzed with chi-square test with Yates’ correction and Student’s *t*-test. For all tests, probability less than 0.05 was considered significant. There were no statistically significant differences between the groups I (AO) and II (AO) and between groups I (WH) and II (WH) with respect to age, sex, time before operation, mode of trauma and diameter of the nails used.

## Results

**Fig. 4:**
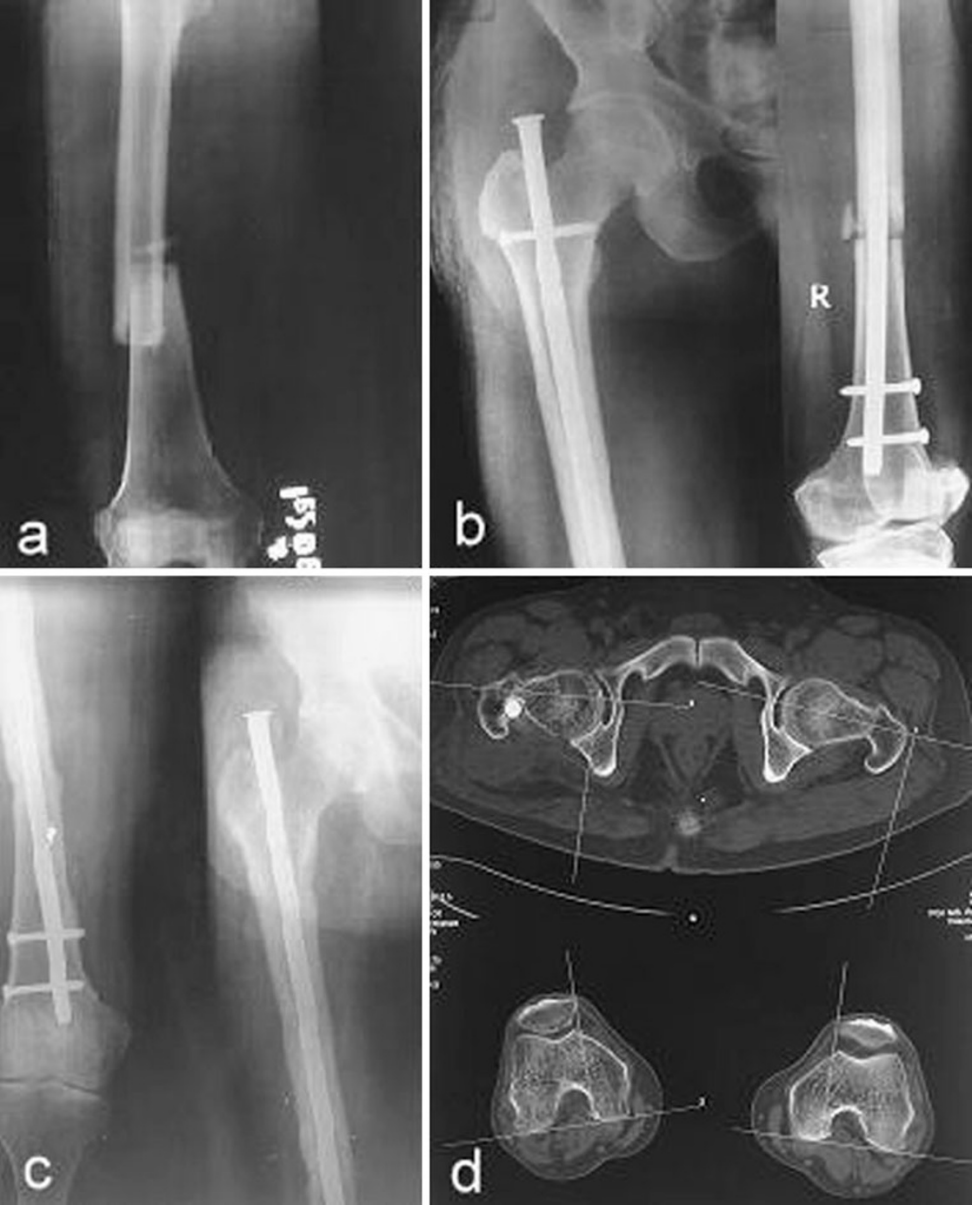
**a** Preoperative anteroposterior radiograph of thigh showing AO type B2 fracture and type I fracture according to Winquist–Hansen classification. **b** Immediate postoperative anteroposterior radiograph showing stabilization of fracture with interlocking nail. **c** Dynamization by removing proximal screw was performed to achieve union. **d** CT scan showed external rotational deformity of 21°

**Fig. 5:**
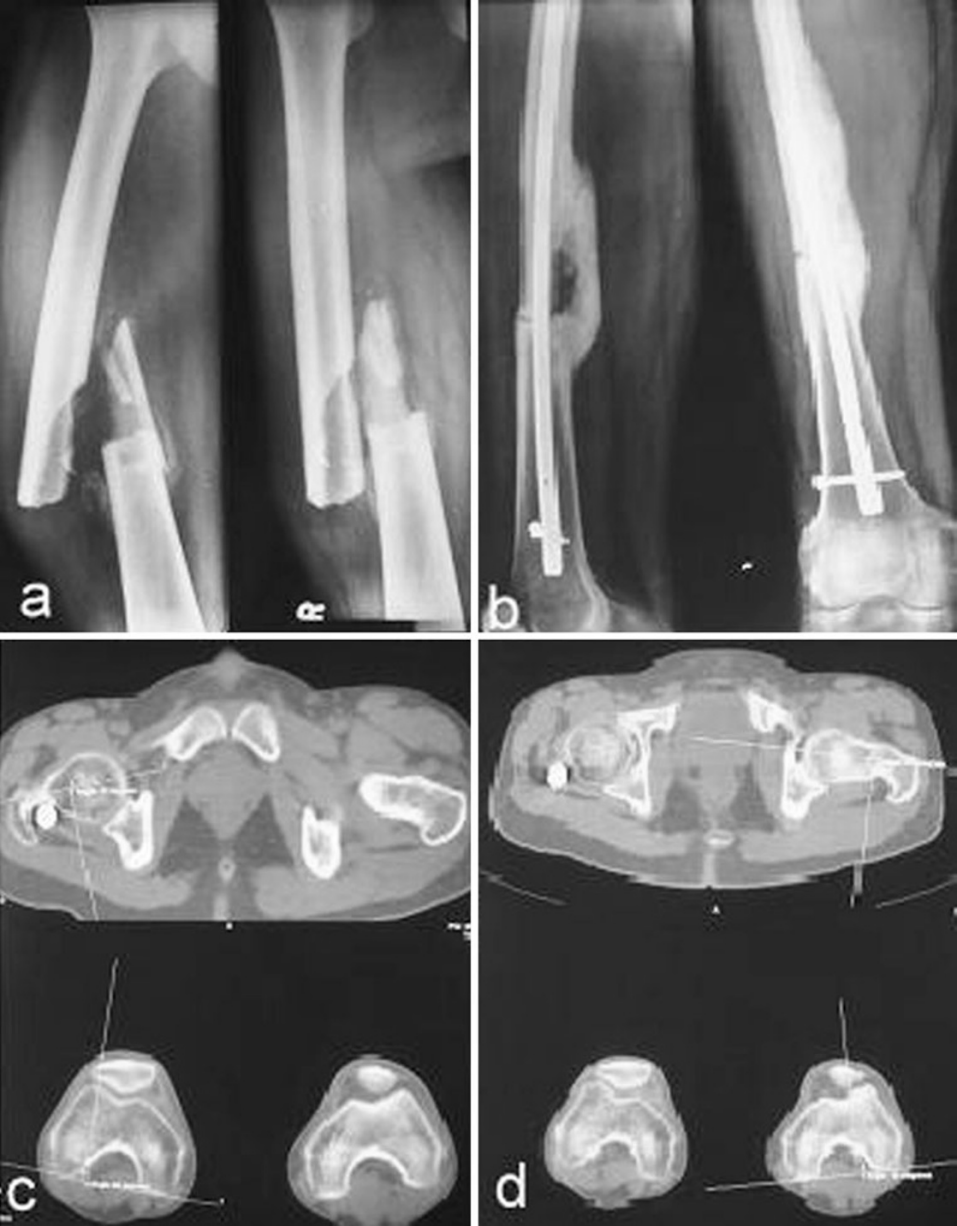
**a** Preoperative anteroposterior and lateral radiographs of thigh showing AO type B3 fracture and type III fracture according to Winquist–Hansen classification. **b** Postoperative anteroposterior and lateral radiographs showing union. **c** and **d** CT scan showed acceptable rotational alignment achieved in the patient (internal rotation deformity of 5°)

Closed nailing was succeeded in 38 patients. Open reduction was required in 3 fractures; one type B fracture and two type C2 fractures as per AO classification; or one type III and 2 type IV fractures according to Winquist–Hansen classification [3]. Schanz screw was used in the distal fragment for closed reduction in 10 patients and in intermediate segmental fragment in 2 patients. Intra-operative displacement of hairline fracture occurred in one patient, but it did not affect the ultimate functional outcome. Average diameter of the nails used was 11.02 mm (range, 9–12 mm). Distal locking was achieved successfully in 37 patients, and the technique failed in 4 patients. Average number of intra-operative radiographic exposures was 4.4 (range, 1–12). Radiation exposures were required either for confirmation of successful insertion of guide wire and distal locking or for interlocking in failures of the technique. The number of radiation exposures was less in group I (AO) (2.92) as compared to that of group II (AO) (5.11), which was statistically very significant (*P* = 0.0053). Similarly, the number of radiation exposures was less in group I(WH) (3.52) as compared to group II(WH) (7.1) which was statistically extremely significant (*P* = 0.0001). Mean operation time was 87.6 min (range, 59–115 min). The mean operative time was more in comminuted fractures, but it was not statistically significant. One nail (9 mm) bent in one patient with AO type B fracture after 4 months of surgery and closed exchange nailing with 11-mm nail resulted in union in this patient. One patient with segmental fracture (AO type C2) stabilized with 10-mm nail developed nonunion at one fracture site and open exchange nailing using a large diameter nail (12 mm) with bone grafting from ipsilateral iliac crest was required to achieve union of the fracture at 13 months. Until the last follow up, no other nail had been removed. Two patients needed dynamization of the fracture to achieve union (Fig. 4a–d). No patient had fracture or infection at interlocking screw and Schanz screw sites. No cases of deep infection and avascular necrosis of femoral head were recorded. Patients were followed up for a mean of 24.3 months (range, 18–30 months). All fractures united at average union time of 19.1 weeks (range, 15–56 weeks) (Fig. 5a–d). A mean limb length shortening of 1.75 cm (range, 1–2.5 cm) was detected in 4 patients. Angular and rotatory malalignments were observed in 7 patients. One patient had 7° varus angulation, and one patient had 9° valgus angulation at fracture site. One patient had both 12° valgus angulation and 15° external rotation deformity, and other four patients had external rotation deformity (mean 20°: range, 15–27) on CT scan measurements. The cumulative complication (including nonunion, limb shortening, angular and rotatory malalignments) rate was 22%.

## Discussion

The femoral shaft fractures in adults are preferably treated with closed intramedullary nailing [1–4] as intramedullary nailing of femoral fractures gives excellent fracture healing, rapid patient recovery and few complications [7]. The fracture union in the present series (97.5%) is comparable with the 92–100% reported in the literature in closed intramedullary nailing [1, 7, 9, 17, 20]. Closed reduction is a critical component of the procedure. At times, closed reduction can be difficult [2], more so in lateral position [23]. Many techniques have been reported to assist closed reduction like use of a small diameter nail in the proximal fragment [3, 21, 27], 8 mm straight reamer into the proximal fragment [17], use of a Schanz pin as percutaneous skeletal joystick in either of the fragments [2]. However, simultaneous use of cannulated reamer in proximal fragment as intramedullary joystick and Schanz screw in the distal fragment as percutaneous joystick has never been reported earlier. The closed nailing without fracture table or C-arm has been reported [17] earlier also, and Aiyer et al. [17] used 8-mm straight reamer in proximal fragment to reduce the fracture. But guide wire needs to be inserted alongside reamer or reamer has to be removed for guide wire insertion, which can lead to loss of reduction. However, in the present technique, cannulation of the reamer allows direct insertion of the guide wire without removal of the reamer. We believe that simultaneous use of cannulated reamer in proximal fragment as intramedullary joystick and Schanz screw in the distal fragment as percutaneous joystick further helps in achieving closed reduction of the fracture and insertion of the guide wire (achieved in 92.6% fractures in the present study). The unicortical nature of the Schanz pin allows for passage of the guide wire. The present technique does not prolong the operative time as the average operative time in the present study is within the range (71–118 min) reported in the literature [4, 9, 11, 17–20, 25].

The rate of angular malalignment in the present series (7.3%) is comparable with the rates (4.5–11.5%) reported in the literature [1, 9, 10, 12, 18, 27]. The rate of limb shortening in the present series (9.7%) is comparable with the rates (4.5–9%) reported in the literature [1, 7, 9]. The many techniques used for intra-operative alignment of the fractures like the cable technique, blumensaat’s line, meterstick technique and lesser trochanter shape sign are image intensifier dependent or need radiographic exposure [28] whereas on an average, only 4.4 radiographic exposures were required to achieve closed locked intramedullary nailing in the present study. However, more radiographic exposures were required in comminuted fractures (*P* value statistically very significant).

Rotational malalignment is a problem with closed intramedullary nailing [7, 22]. The rate of rotational malalignment in the present series (12.2%) is comparable with the rates (0–28%) reported in the literature [1, 6, 7, 9, 12, 13, 17, 26]. Studies using just clinical assessment reported a very low or no incidence of malalignment [9, 12, 17, 23, 27] but accuracy of a clinically determined rotational malalignment is poor compared with a CT-determined rotational malalignment [26]. We have used postoperative CT scan to assess malrotation as it is a reliable and more reproducible and therefore the preferred method [26, 28]. Now, we are not using CT scan in every patient. This was used only to standardize the present technique. We have used patella as parallel to the floor for lateral nailing in the present study. Other studies have also reported this method to achieve rotational alignment [17, 22], whereas Winquist et al. [3] assessed malrotation through careful attention to the skin folds. Control of rotational alignment seems to be more difficult in lateral position [13]. We have used padded posts on sacrum and anterior superior iliac spines to stabilize the pelvis, and this might have prevented secondary effect of pelvis mal-position. The padded posts are not costly and can be fabricated locally.

Open reduction was required in 7.3% fractures in the present study, which is comparable with 4–10% reported in the literature [9, 11, 17]. In the present study, all fractures needing open reduction were comminuted or segmental fractures. We advise against reaming without confirming the intramedullary position of the guide wire in distal fragment. We advise open reduction through a mini-wound at fracture site in case of difficulty, and this method has also been reported to achieve satisfactory healing [19]. The technique failed to achieve distal locking in 10% patients in the present study. Failure of the distal locking jig has been reported in other series also [17]. Over reaming of the medullary canal by 1.5–2 mm has been advised to prevent nail deformation during insertion [8, 17, 25].

## Conclusion

We are of the opinion that the present technique is a safe and reliable alternative to achieve closed locked intramedullary nailing without the use of image intensifier and fracture table. It is best suited to stable, less comminuted (Winquist–Hansen types I and II) diaphyseal fractures of the femur although the technique can be used in severely comminuted fractures.
